# Formulation of a killed whole cell pneumococcus vaccine - effect of aluminum adjuvants on the antibody and IL-17 response

**DOI:** 10.1186/1476-8518-9-5

**Published:** 2011-07-29

**Authors:** Harm HogenEsch, Anisa Dunham, Bethany Hansen, Kathleen Anderson, Jean-Francois Maisonneuve, Stanley L Hem

**Affiliations:** 1 Department of Comparative Pathobiology, Purdue University, 725 Harrison Street, West Lafayette, IN 47907, USA; 2 PATH, Seattle, WA, USA; 3 Department of Industrial and Physical Pharmacy, Purdue University, IN, USA

## Abstract

**Background:**

*Streptococcus pneumoniae *causes widespread morbidity and mortality. Current vaccines contain free polysaccharides or protein-polysaccharide conjugates, and do not induce protection against serotypes that are not included in the vaccines. An affordable and broadly protective vaccine is very desirable. The goal of this study was to determine the optimal formulation of a killed whole cell pneumococcal vaccine with aluminum-containing adjuvants for intramuscular injection.

**Methods:**

Four aluminium-containing adjuvants were prepared with different levels of surface phosphate groups resulting in different adsorptive capacities and affinities for the vaccine antigens. Mice were immunized three times and the antigen-specific antibody titers and IL-17 responses in blood were analyzed.

**Results:**

Although all adjuvants induced significantly higher antibody titers than antigen without adjuvant, the vaccine containing aluminum phosphate adjuvant (AP) produced the highest antibody response when low doses of antigen were used. Aluminum hydroxide adjuvant (AH) induced an equal or better antibody response at high doses compared with AP. Vaccines formulated with AH, but not with AP, induced an IL-17 response. The vaccine formulated with AH was stable and retained full immunogenicity when stored at 4°C for 4 months.

**Conclusions:**

Antibodies are important for protection against systemic streptococcal disease and IL-17 is critical in the prevention of nasopharyngeal colonization by *S. pneumoniae *in the mouse model. The formulation of the whole killed bacterial cells with AH resulted in a stable vaccine that induced both antibodies and an IL-17 response. These experiments underscore the importance of formulation studies with aluminium containing adjuvants for the development of stable and effective vaccines.

## Background

*Streptococcus pneumoniae *(pneumococcus) is a Gram-positive, encapsulated diplococcus that is commonly present as a commensal bacterium in the microbial flora of the upper respiratory tract without causing clinical disease. However, these bacteria also cause great morbidity and mortality throughout the world. Pneumococcal infections are a leading cause of pneumonia, bacteremia, meningitis, and otitis media in adults and children, and account for an estimated 1.6 million deaths, including up to 1 million children less than 5 years of age, annually [1-3]. The burden of disease is greatest in developing countries.

Based on differences in the composition of the polysaccharide capsule, more than 90 distinct serotypes of pneumococcus are recognized. Current vaccines against pneumococcus are a 23-valent vaccine containing free polysaccharides and 7-valent, 10-valent and 13-valent vaccines composed of protein-polysaccharide conjugates. The free polysaccharides are T-independent antigens and induce a poor immune response in children less than 2 years of age. In contrast, the conjugated vaccines that are T-dependent induce a good immune response in young children and infants. These vaccines have greatly reduced disease caused by the pneumococcal serotypes included in the vaccines in countries where these vaccines are widely used. However, the vaccines do not protect against serotypes that are not included in the vaccine. Many serotypes in developing countries are not included in the currently available vaccines and widespread adoption of the vaccines is limited by the cost of the polysaccharide and conjugate vaccines. Furthermore, increased prevalence of non-vaccine serotypes has been observed following the implementation of pneumococcus vaccination programs [4,5]. These considerations have led to the pursuit of alternative vaccination strategies, including the use of protein antigens that are shared among the different serotypes. A potentially successful approach is the use of killed, non-encapsulated pneumococci (whole cell antigen - WCA) which provides multiple common antigens for inducing an immune response that is protective across the different serotypes, and is relatively inexpensive to prepare [6].

Previous studies showed that intranasal immunization with WCA and cholera toxin as a mucosal adjuvant, induced a robust antibody response [7]. The inoculated mice had greatly reduced nasopharyngeal and middle ear colonization following intranasal administration of pneumococci of different serotypes [7-9]. Similarly inoculated rats were protected from sepsis against intrathoracic challenge with serotype 3 [7]. The protection against nasopharyngeal colonization in mice occurred in antibody-deficient mice, and was dependent on the presence of CD4^+ ^T cells. Subsequent studies demonstrated that this protection was conferred by Th17 cells, whereas IL-4 and IFN-γ were not necessary for protection [10].

Although mucosal administration of vaccines has several advantages, the need for cholera toxin to induce an effective immune response precludes this route of immunization for human use until acceptable mucosal adjuvants become available. Vaccines for intramuscular injection often contain aluminum compounds as safe, effective, and inexpensive adjuvants. The two aluminum-containing adjuvants that are commercially available and widely used in vaccines are aluminum hydroxide (AH) and aluminum phosphate (AP) [11]. These adjuvants have large adsorptive surfaces, but different structural and surface properties which affect their interaction with vaccine antigens. Adsorption of antigens onto aluminum adjuvants increases the retention of antigens at the injection site and this property was considered essential for the immunostimulatory effect ("depot-mechanism"). However, recent studies indicate that adsorption is not necessary for the adjuvant effect of aluminum compounds [12-14]. Nevertheless, adsorption may affect the structural stability of antigens and the availability of epitopes [15,16]. The two main mechanisms by which antigens adsorb onto aluminum-containing adjuvants are electrostatic attraction and ligand exchange [11]. The surface charge of AH is positive at neutral pH and that of AP is negative at neutral pH. Therefore, these adjuvants have different affinities for antigens that adsorb through electrostatic mechanisms. Electrostatically adsorbed antigens usually elute from the adjuvants upon exposure to interstitial fluid following intramuscular or subcutaneous administration [17]. Ligand exchange is the replacement of surface hydroxyls by terminal phosphate groups of phosphorylated antigens creating a covalent bond that is stronger than electrostatic adsorption. Since AH has more surface hydroxyls than AP, it has a higher affinity for phosphorylated antigens. Such strong adsorption results in poor elution in interstitial fluid and has a negative effect on the immune response to phosphorylated antigens formulated with AH as opposed to AP [18].

Our previous work with aluminium-containing adjuvants was based on single antigens. Here, we report on experiments aimed at formulating WCA, a complex mixture of antigens, with aluminum adjuvants. The goal was to determine the formulation that induced the maximum antibody and IL-17 response, two critical components of a protective immune response against *S. pneumoniae *[6]. These studies for the first time demonstrate that the type of aluminum-containing adjuvants (AH vs. AP) affects the magnitude and quality of the antibody response as well as the Th17 CD4^+ ^T cell response to WCA.

## Methods

### Mice

All experiments involving mice were conducted in accordance with NIH guidelines for the care and use of experimental animals and were approved by the Purdue University Animal Care and Use Committee. Seven week old female C57BL/6J mice were purchased from the Jackson Laboratory (Bar Harbor, ME). Mice were maintained in a conventional barrier facility, exposed to a 12 h light/12 h dark cycle, and allowed free access to water and LabDiet 5015 (Purina Mills, Richmond, IN, USA). They were acclimated for one week, and injected with 50 μl of vaccine intramuscularly in each hind leg (100 μL/mouse) two or three times with a two-week interval. Immediately prior to the last injection, blood was collected from the facial vein. Two weeks after the last injection, mice were anesthetized, blood was collected in heparinized tubes, and the mice were euthanized. Serum and plasma were separated by centrifugation at 14,000 × g for 10 min and stored at -80°C until analysis.

### Vaccine preparations

The whole cell bacterial antigen (WCA) consists of a suspension of strain Rx1E, a capsule-deficient, autolysin-negative mutant of *Streptococcus pneumoniae*, killed by treatment with beta-propiolactone [19]. The stock solution (prepared by Instituto Butantan, Sao Paulo, Brazil) contained 10^10 ^cells/mL (corresponding with 10 mg protein/mL) in Ringer's solution.

Vaccines were prepared with 4 different adjuvants. Aluminum hydroxide adjuvant (Alhydrogel "85" 2%) and AP (AdjuPhos) were obtained from Brenntag Biosector (Denmark). Phosphate-treated AH (PTAH) and phosphate treated AP (PTAP) were prepared by mixing the adjuvants with 60 mM phosphate buffer for 16 hours at room temperature.

Vaccines were prepared aseptically by adding different amounts of WCA as indicated in the text to adjuvants at 1.2 mg Al/mL and mixing for 1 h at room temperature.

### Adsorption isotherms

Vaccines were prepared as described above with different WCA concentrations. After incubation for 1 h at 4°C, the suspension was layered over a 60% sucrose gradient and centrifuged for 20 minutes at 1,500 × g to separate non-adsorbed WCA from adsorbed WCA. The supernatant was collected and protein content was determined by bicinchoninic acid protein assay (Pierce, Rockford, IL) in triplicate. The adsorption data was plotted according to the linear form of the Langmuir equation. The adsorptive coefficient was calculated as the slope/intercept and the adsorptive capacity was calculated as the reciprocal of the slope.

### Light microscopy of vaccine preparations

The bacterial cells in WCA were stained with gentian violet prior to mixing with AH and AP. The stained cells were mixed with each adjuvant and examined by light microscopy using a 100× oil immersion objective.

### Anti-WCA ELISA

Ninety-six well plates were coated with WCA (10^8^/mL) overnight, blocked with 5% fetal calf serum diluted in PBS, and incubated with serially diluted standard and serum or plasma samples starting at a 1:100 dilution. The plates were then incubated with peroxidase-labeled goat anti-mouse IgG (Sigma, St. Louis, MO), followed by 3,3',5,5' - tetramethylbenzidine substrate. After addition of a 2 N sulfuric acid stop solution the color intensity was measured in a microplate reader (Biotek, Winooski, VT) at 450 nm. A standard curve was constructed using serum with high antibody titer, arbitrarily set at 120,000 U/mL.

### Immunoblot of plasma samples

The WCA was diluted to 10^9^/mL in lithium dodecyl sulfate (LDS) sample buffer (Thermo Fisher Scientific, Rockford, IL) and incubated for 10 minutes at 70°C. The proteins were separated on a 4-12% gradient gel (Invitrogen) and transferred onto nitrocellulose. Individual strips were blocked with non-fat milk, incubated with pooled plasma at 1:500 dilution from each of the experimental groups, and then with peroxidase-labeled goat anti-mouse IgG. Bands were visualized with an ECL detection kit.

### IL-17 assay

Forty microliters of heparinized blood was added to 360 μL of Iscove's Modified Eagle Medium supplemented with 10% fetal calf serum, 10 μg/mL ciprofloxacin, and 10^7 ^WCA/mL. After incubation for 6 days at 37°C and 5% CO_2_, supernatants were collected and stored at -80°C until analysis by ELISA for IL-17A (IL17; R&D Systems, Minneapolis, MN).

### Statistical analysis

The anti-WCA IgG concentrations were log2 transformed prior to analysis by one-way ANOVA followed by a Newman-Keuls multiple comparison test (Graphpad Prism, version 5.02). Differences between groups at p < 0.05 were considered significant. The statistical significance of differences between means of IL-17 among experimental groups was determined by two-way ANOVA followed by Bonferroni post-hoc test with p < 0.05.

## Results

### Adsorption of WCA onto aluminum-containing adjuvants

**Figure 1:**
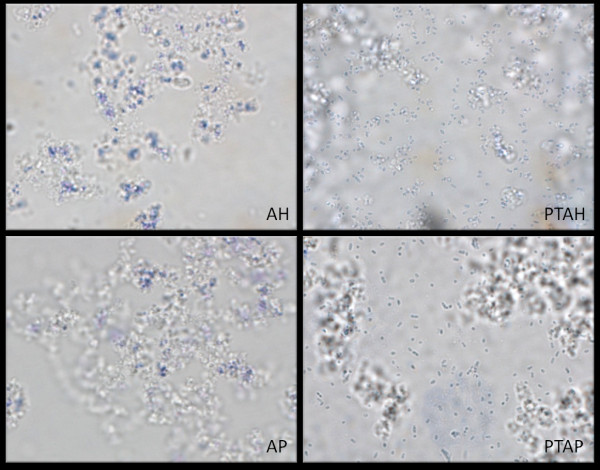
**Phosphate treatment of aluminum hydroxide adjuvant and aluminum phosphate adjuvant prevented the adsorption of bacterial pneumococcal cells**. Light microscopy of WCA mixed with the aluminum-containing adjuvants, aluminum hydroxide adjuvant (AH), aluminum phosphate adjuvant (AP), phosphate-treated AH (PTAH) and phosphate-treated AP (PTAP). The bacteria were stained with gentian violet, and the suspensions were examined using a 100× oil objective.

Four different adjuvants were prepared and incubated with different doses of WCA to determine the adsorptive capacity and coefficient. The adsorptive capacity and adsorptive coefficient (adsorptive strength) of AH was greatest, followed by AP and then PTAH (Table 1). There was no detectable adsorption of protein to PTAP. The adsorption of the bacterial cells to each adjuvant was verified by light microscopy using gentian violet-stained bacteria (Figure 1). The bacteria were associated with the AH and AP aggregates and were not observed in the liquid phase separating the adjuvant aggregates. In the case of PTAH and PTAP, the bacteria were largely present in the liquid regions. These bacteria were moving freely by Brownian motion while the cells associated with the AH and AP adjuvant aggregates were stationary. Thus, the observations by light microscopy concurred with the data derived from the adsorption isotherms (Table 1).

**Table 1:** Adsorptive capacity and adsorptive coefficient (affinity) of the different adjuvants for WCA calculated from Langmuir adsorption isotherms.

Langmuir isotherm coefficient	WCA/AH	WCA/AP	WCA/PTAH	WCA/PTAP
Adsorptive capacity (mg/mg Al)	0.22	0.07	0.03	- ^a^
Adsorptive coefficient (mL/mg)	4500	2026	803	- ^a^

^a ^Adsorptive capacity and coefficient could not be determined because there was no detectable adsorption.

### Antibody response to vaccines formulated with four different adjuvants

**Figure 2:**
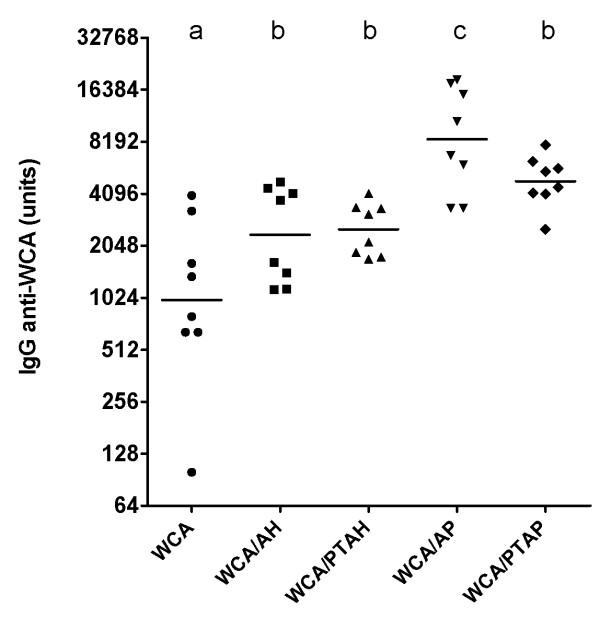
**IgG titers in mice injected with vaccines with different types of aluminium-containing adjuvants**. Mice (n = 8/group) were injected twice with WCA (10^7 ^cells/dose) alone or combined with four different aluminum-containing adjuvants. The IgG titer in serum was determined two weeks after the second injection. The symbols represent individual mice and the horizontal line indicates the geometric mean. The geometric means of groups with different letters are different at p < 0.05.

Mice were injected with WCA (10^7 ^cells) alone or combined with one of the four adjuvants. After two injections, blood was collected and the concentration of anti-WCA IgG was determined. All four adjuvants enhanced the antibody response over WCA alone. The highest concentration of anti-WCA IgG was observed in mice injected with WCA/AP, followed by WCA/PTAP, WCA/PTAH, and WCA/AH (Figure 2). The difference between WCA/AP and WCA/AH was statistically significant.

### Effect of AH vs. AP on antibody and IL-17 responses

**Figure 3:**
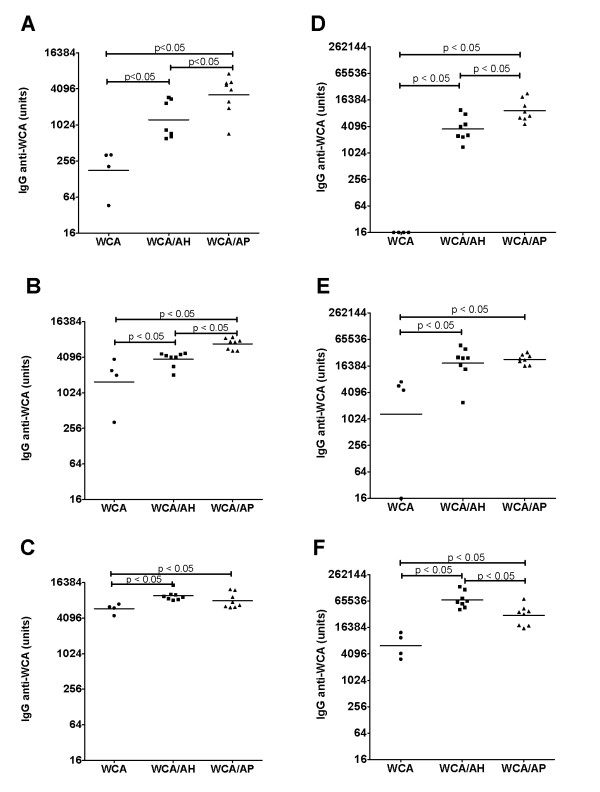
**IgG titers in mice injected with vaccines formulated with AH and AP and different doses of WCA**. Mice were immunized twice with WCA at 10^6 ^cells (A), 10^7 ^cells (B) and 10^8 ^cells (C) per dose, or three times with WCA at 10^6 ^cells (D), 10^7 ^cells (E) and 10^8 ^cells (F) per dose. The symbols represent individual mice (n = 4/group for WCA alone and n = 8 for WCA/AH and WCA/AP) and the horizontal line indicates the geometric mean.

Since phosphate treatment of the AH and AP adjuvants did not enhance the immunostimulatory effect of these adjuvants, subsequent experiments were conducted with AH and AP only. Mice were injected with 3 different doses of WCA alone or combined with AH or AP. Blood was collected after two and three injections for the determination of anti-WCA antibody concentrations, and after three injections for IL-17 production. The adjuvants significantly enhanced the antibody response to WCA at all three doses and after two as well as three immunizations (Figure 3). The anti-WCA IgG concentration generally increased with increasing dose and after more immunizations. At the lowest dose of WCA (10^6 ^cells), the mice that received WCA/AP generated a stronger antibody response than mice injected with WCA/AH. At the intermediate dose (10^7 ^cells), WCA/AP induced a stronger antibody response after two injections, while there was no difference between the WCA/AP and WCA/AH groups after three injections. There was also no difference between WCA/AP and WCA/AH after two injections of the highest dose (10^8 ^cells), but after three injections the mice that received WCA/AH had the highest IgG concentration. Previous experiments showed that anti-WCA IgG concentrations > 10,000 units/mL are protective upon challenge in mice [19]. These values were consistently obtained after three injections with 10^7 ^and 10^8 ^cells when formulated with AP, and with 10^8 ^cells when formulated with AH. Thus, the effect of aluminum-containing adjuvants is dose-dependent with AP generating a stronger antibody response at lower antigen doses.

**Figure 4:**
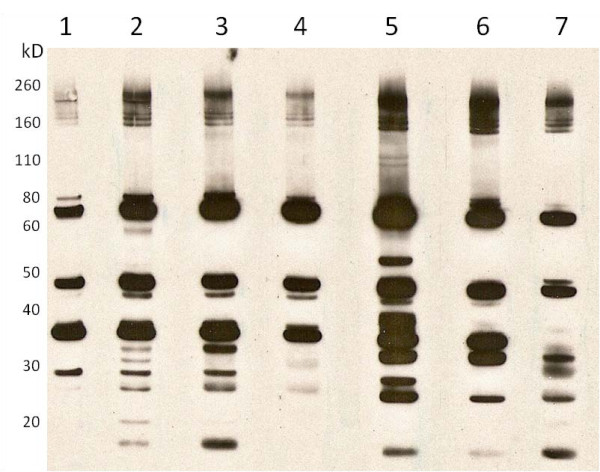
**Antigen specificity of IgG from mice injected with vaccines formulated with AH and AP**. Immunoblot of WCA with antibodies in pooled plasma from mice injected three times with WCA only at 10^8^/dose (lane 1); WCA/AP at 10^8^/dose (lane 2), 10^7^/dose (lane 3), 10^6^/dose (lane 4); and WCA/AH at 10^8^/dose (lane 5), 10^7^/dose (lane 6), 10^6^/dose (lane 7). Plasma was collected 2 weeks after the last injection.

The adjuvants AH and AP have opposite surface charges at pH 6-7 resulting in different affinities for proteins with different isoelectric points. To determine if these differences affect which WCA proteins induce an antibody response, an immunoblot was performed with WCA as substrate and pooled plasma from mice in each of the vaccine groups (Figure 4). The antibodies reacted with a range of proteins varying in size from less than 20 kD to over 200 kD. Consistent with the ELISA results, the bands from mice immunized with the highest dose of WCA in combination with AH had the greatest intensity. Antibodies from mice injected with adjuvanted WCA reacted with more proteins than antibodies from mice injected with WCA only. In addition, there were several proteins in the 30 - 60 kD range that reacted only with antibodies from mice immunized with AH or with AP-adjuvanted vaccines (Figure 4).

**Figure 5:**
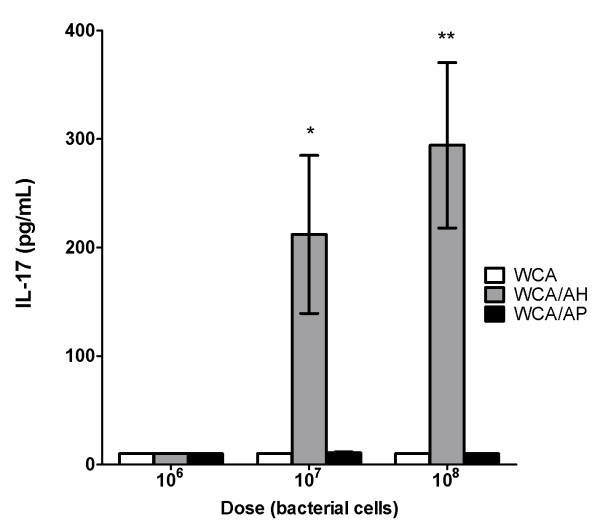
**Vaccines of WCA formulated with AH, but not with AP or vaccines without adjuvants induced an IL-17 response**. IL-17 concentration in the supernatant of whole blood cultures incubated for 6 days with WCA (10^7^/mL). The blood was collected from mice injected three times with WCA, WCA/AH or WCA/AP at 10^6 ^cells/dose, 10^7 ^cells/dose or 10^8 ^cells/dose. The bars indicate the mean ± SEM of 8 mice per group. * p < 0.05; ** p < 0.005 (WCA/AH vs. WCA and WCA/AH vs. WCA/AP).

The concentration of IL-17A (IL-17) was determined in the supernatant of whole blood cultures following incubation with WCA for 6 days. A significant concentration of IL-17 was only detected in cultures from mice injected with the intermediate and high dose of WCA in combination with AH. There was no detectable IL-17 in blood cultures from any of the other groups (Figure 5).

### Stability of the WCA/AH vaccine formulation

**Figure 6:**
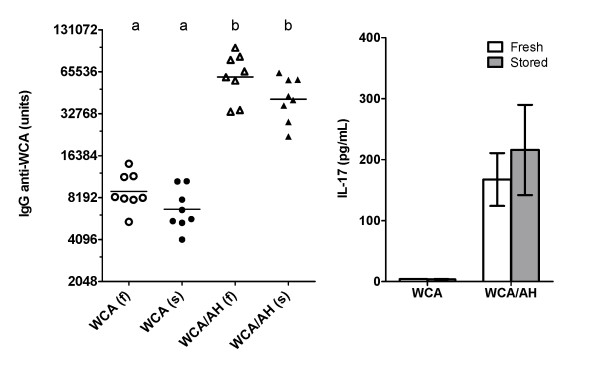
**Immunogenicity of the WCA/AH vaccine formulation stored for 4 months at 4°C**. Mice were immunized three times with WCA (10^8 ^cells/dose) and WCA/AH stored at 4°C for four months (s) or with freshly prepared WCA and WCA/AH (f). The IgG titer was determined in plasma collected 2 weeks after the last injection. The IL-17 concentration was determined in the supernatant of whole blood cultures stimulated with WCA.

To determine the effect of prolonged storage of the WCA/AH vaccine on the immune response, the high dose of WCA (10^8 ^cells/dose) was prepared with or without AH and stored for 4 months at 4°C. Mice were injected 3 times with stored and freshly prepared vaccines and the immune response was analyzed as described above. A greater IgG response was observed after three compared with two injections. The IgG response obtained with the stored vaccine formulation was slightly lower than that obtained with the freshly prepared formulation (geometric mean of freshly prepared WCA was 9,073 vs. 6,754 for stored WCA; geometric mean of freshly prepared WCA/AH was 60,256 vs. 41,688 for stored WCA/AH), but the difference was not statistically significant (Figure 6A). Importantly, the IgG titers in mice immunized with the stored vaccine were well above the minimum protective level of 10,000 units/mL. There was no difference in the concentration of IL-17 in supernatants of whole blood cultures of mice immunized with WCA/AH (Figure 6B).

## Discussion

Vaccines against pneumococcal disease for use in developing countries should be safe, effective against a broad range of serotypes and affordable. The existing conjugate vaccines offer protection against the serotypes included in the vaccine which were selected based on their prevalence in North America and Europe, and are predicted to provide incomplete protection against pneumococcal infections in Asia and Africa. In addition, these conjugate vaccines are expensive to produce. The work in this report demonstrates that a vaccine composed of killed whole cell, nonencapsulated pneumococci and formulated with AH, induces a strong antibody and IL-17 response. Both the antigen and adjuvant are relatively inexpensive suggesting that the vaccine will be affordable for use in developing countries.

Previous work with a simple protein antigen, alpha casein, indicated that the strength of adsorption of antigens onto aluminum-containing adjuvants is inversely related to the antibody response to these antigens [18]. A similar relationship was found with a larger and more complex antigen, hepatitis B surface antigen (HBsAg), but the negative effect of a high adsorptive coefficient was not as strong as with alpha casein [20]. The antigen used in the current studies, WCA, consists of killed whole bacterial cells and some soluble bacterial proteins. WCA was mixed with four aluminum-containing adjuvants with different surface properties to determine if differences in adsorptive capacity and adsorptive coefficient could be measured. Although the obtained values should be interpreted with caution because of the complex nature of WCA, they indicate a range of adsorptive properties for the four adjuvants. The highest values were measured for AH while adjuvants with more surface phosphates had a lower affinity for WCA. This suggests that at least some of the molecules in WCA are phosphorylated or associated with phospholipid membranes, and are adsorbed by the ligand exchange mechanism.

The antibody response to the vaccine formulations with the four adjuvants with broadly divergent adsorptive capacities and coefficients for WCA indicated that the aluminum-containing adjuvant potentiated the immune response even when the antigen was not adsorbed. In addition, the strength of adsorption was not a significant factor in immunopotentation. Aluminum adjuvants may enhance the immune response to soluble antigens by adsorbing the antigens onto the adjuvant particles that are more readily phagocytised by antigen-presenting cells [21]. Antigen adsorption by the adjuvant may be less relevant when the antigen comprises killed whole cell bacteria as the bacteria are about 1 micrometer in diameter while the primary particles of the adjuvant are smaller than 50 nm [11]. Since changes in adsorption through phosphate treatment of the adjuvants did not affect the antibody response, subsequent experiments focused on AH and AP.

The protective immune response induced by conjugate vaccines is based on serotype-specific anti-polysaccharide antibodies. In contrast, the immune response against WCA involves antibodies directed against protein antigens and Th17 cells. Antibodies induced by WCA can provide protection against systemic disease, but they do not protect against nasopharyngeal colonization in mice [7,9]. Nasopharyngeal colonization was inhibited by CD4^+ ^T cells that secrete IL-17, and the concentration of IL-17 in WCA-stimulated whole blood cultures was inversely correlated with the degree of nasopharyngeal colonization following intranasal challenge [10]. Infection of naïve mice with *S. pneumonia *induced Th17 cells which provided enhanced clearance of the bacteria upon secondary challenge [22]. The protective role of IL-17 resides in the induction of secretion of antimicrobial peptides and chemokines that attract monocytes and neutrophils to the site of infection [23,24]. IL-17 is also involved in the protection against other extracellular bacterial pathogens such as *Bordetella pertussis*, intracellular bacterial pathogens including *Mycobacterium tuberculosis*, and fungal pathogens, indicating an important role against infections at mucosal surfaces and in the lung. However, an excessive IL-17 response may be detrimental and cause extensive tissue damage [23,24]. It has been suggested that Th17 cells are critical for vaccine-induced memory immune responses, and enhancing and regulating the Th17 response may be important in vaccine design [24]. In our studies, the combination of WCA with AH was critical for the induction of a population IL-17 producing cells following intramuscular injection. Neither WCA alone nor WCA with AP induced a significant IL-17 response, even though AP greatly enhanced the antibody response to WCA. Such a dramatic difference in the quality of the immune response between vaccines formulated with AH and vaccines formulated with AP was unexpected.

The induction of Th17 cells in *S. pneumoniae *infection is dependent on TLR2 [22]. The ligands for TLR2 include molecular components of Gram-positive bacteria such as lipoproteins [25,26]. The induction of Th17 cells by WCA/AH and not by WCA/AP suggests that these ligands are not available in the WCA/AP formulation, possibly due to strong electrostatic adsorption.

There are few published reports in which the immune responses to bacterial vaccines formulated with AP vs. AH are directly compared. In one study, acellular pertussis antigens combined with AH induced a stronger antibody response and greater protection upon intranasal challenge with *Bordetella pertussis *compared with AP, but the basis of the increased protection was not further investigated [27]. Th17 cells are induced during infection with *Bordetella pertussis*, but antibody-mediated depletion of IL-17 only had a modest effect on the bacterial loads in the lungs of experimentally infected mice [28]. Two types of vaccines, a whole cell and an acellular pertusiss vaccine, are used to protect against whooping cough. Both vaccines are effective, but vaccination of mice with a whole cell pertussis vaccine induced Th17 cells, whereas these cells were not induced by the acellular vaccine [29,30]. The role of adjuvants was not specifically addressed in these studies.

The basis for the difference in immune response generated by WCA formulated with AH vs. AP is not entirely clear, but it is likely that the greater affinity of AH for WCA proteins contributed to this effect. The adsorptive strength, determined as the adsorptive coefficient, of AH was 2.5 times that of AP. Previous work showed that a high adsorptive strength may interfere with the antibody response and the T cell response, probably because there is insufficient release of antigen from the adjuvant to interact with B cells and for antigen processing and presentation [18]. A similar effect was observed at the lower doses of WCA in which a significantly stronger antibody response was obtained with AP in comparison with AH. At higher doses, the difference between AP and AH disappeared and AH induced a stronger antibody response than AP at the highest antigen dose.

Immunoblot analysis revealed qualitative and quantitative differences in the antigenic proteins recognized by antibodies from the mice injected with different WCA formulations. The antibodies from mice injected with adjuvanted WCA reacted with more proteins than those from mice injected with non-adjuvanted WCA. Antibodies from mice injected with WCA/AH and WCA/AP reacted with an overlapping, but different set of proteins. The surface of AH and AP have opposite charges at pH 6-7 resulting in different affinities for individual proteins within the WCA. This may in turn affect which antigens from this complex protein mixture induce antibodies. Further studies are necessary to determine the biological significance of these differences in antibody specificities.

Long term stability of vaccines is an important consideration. In order to assess the stability of the WCA/AH vaccine formulation, the effect of prolonged storage at 4°C on the immune response was determined. There was no significant difference between the stored and freshly prepared formulations indicating that the WCA/AH is quite stable.

## Conclusions

The goal of these experiments was to determine the optimal formulation of a killed pneumococcal vaccine with aluminium-containing adjuvants. The data indicate that formulation of WCA with AH induces a strong antibody and Th17 response, and AH is the preferred choice over AP for vaccines for intramuscular administration. The marked differences in the antibody and cellular response to the two aluminum-containing adjuvants underscores the importance of proper pre-formulation studies in preparing safe and effective vaccines [31,32].

## Competing interests

The authors declare that they have no competing interests.

## Authors' contributions

HH and AD carried out the mouse experiments, and BH did the adsorption experiments. AD and KA performed the immunoassays. HH, JFM and SLH designed the study. HH and SLH coordinated the experiments and wrote the manuscript. The manuscript was reviewed and approved by all authors.
